# A cross-sectional study of infant feeding practices in Vietnamese-born mothers living in Australia

**DOI:** 10.1186/s12884-022-05223-8

**Published:** 2022-12-03

**Authors:** Lauren Zahra, Peter Kremer, Kristy A. Bolton

**Affiliations:** 1 School of Exercise and Nutrition Sciences, Deakin University, Burwood, VIC Australia; 2 Centre for Sport Research, School of Exercise and Nutrition Sciences, Deakin University, Geelong, VIC Australia; 3 Institute for Physical Activity and Nutrition, School of Exercise and Nutrition Sciences, Deakin University, Geelong, VIC Australia

**Keywords:** Early childhood, Breastfeeding, Ethnicity, Immigrants, Culture, Vietnamese, Obesity, Maternal child health

## Abstract

**Background:**

Infant feeding practices are a key modifiable risk factor for childhood overweight and obesity; and important for lifelong health and wellbeing. Despite the growing Australian immigrant population, it is unclear how infant feeding practices may differ between ethnicities living in Australia. Few studies have examined the infant feeding practices of Vietnamese mothers who migrate and give birth to infants in Australia – termed *Vietnamese-born mothers*. The aim of this study was to examine differences in infant feeding practices (breastfeeding, formula feeding and complementary feeding (other fluids and solids)) in Vietnamese-born mothers compared with Australian-born mothers living in Australia.

**Method:**

This study analysed the Australian National Infant Feeding Survey dataset (2010–11), a large national cross-sectional survey measuring feeding practices of infants aged 0–24 months old. Infant feeding practices of Vietnamese-born mothers (*n* = 261) and a random sub-sample of Australian-born mothers (*n* = 261) were compared. Associations between ethnicity and infant feeding practices were examined through logistic and linear regression adjusting for maternal age, socioeconomic status, body mass index (BMI) at start of pregnancy, infant age at survey completion and parity. Compliance with the Australian national infant feeding guidelines was also assessed.

**Results:**

Compliance with infant feeding guidelines was low, with differences in infant feeding practices between groups. At the time of survey completion, when infants were on average 7.2 months old, compared with infants of Australian-born mothers, infants to Vietnamese-born mothers were significantly younger when first exposed to fruit juice (*b* = -2.41, 95%CI: -4.54– -0.28); less likely to be exposed to solids (AOR: 0.15, 95%CI: 0.05–0.44) and more likely to be exposed to formula milks (AOR: 2.21, 95%CI: 1.10–4.43); toddler milks (AOR: 16.72, 95%CI: 3.11–90.09) and fruit juice (AOR: 2.37, 95%CI: 1.06–5.32) (*p* < 0.05).

**Conclusion:**

Low adherence with breastfeeding (low breastfeeding and high infant formula use) and other fluids (toddler milks and fruit juice) recommendations outlined by the Australian infant feeding guidelines were observed in this group of Vietnamese-born mothers. To optimise feeding and growth in Vietnamese-Australian children, culturally appropriate infant feeding support targeting breastfeeding durations, reducing reliance on infant formula, and reducing inappropriate introduction to other fluids should be the focus of infant feeding promotion within these mothers.

**Supplementary Information:**

The online version contains supplementary material available at 10.1186/s12884-022-05223-8.

## Background

Infant feeding practices within the first one thousand days of life is a critical period for the healthy growth and development of an infant [1–3]. Infant feeding practices refer to breastfeeding, formula feeding, and complementary feeding [4, 5]. The World Health Organization recommends exclusive breastfeeding for the first six months of an infant’s life, after which complementary foods such as solids are introduced, and breastfeeding should continue until 12 months of age in combination with solid foods [4, 5].

The increasing prevalence of childhood overweight and obesity is a global health priority [6]. Despite the multifaceted nature of childhood overweight and obesity, evidence indicates infant feeding practices play a key role in weight gain trajectories during infancy [7–9]. Excess or rapid weight gain in this time, has been associated with subsequent childhood adiposity [10, 11]. This indicates that emphasis should be placed on adherence to recommended infant feeding practices during childhood [4].

Globally, significant increases in immigration to higher socioeconomic countries have been observed over the last decade [12]. Existing literature shows ethnicity is a predictor of infant feeding practices [13–16]. International studies show Indian, Pakistani, Black Caribbean, Black African and Asian mothers demonstrate health promoting infant feeding practices such as longer breastfeeding durations [17] and appropriate timing of introduction of solids compared with their white/native-born counterparts [15].

Approximately 30% of the Australian population are immigrants [18]. South, Central, South-East and North-East Asian regions account for a growing proportion of incoming migrants entering Australia [18]. Research of infant feeding practices in Asian sub-groups living in Australia is scarce and outdated. Recently, it has been revealed that Chinese-born immigrant mothers living in Australia have different infant feeding practices compared with Australian-born counterparts; including low adherence to the Australian infant feeding guidelines [19] and infants of Chinese-born immigrant mothers have rapid weight gain trajectories during early infancy [20].

Vietnamese immigrants are the fifth largest immigrant group living in Australia [18]. Few studies have examined a wide range of infant feeding practices in Vietnamese-born mothers living in Australia in isolation to other Asian ethnic groups [14, 21–23]. By combining Asian ethnic groups (which typically have different cultures, traditions, religions and languages) into an overarching ‘umbrella’ group, subtle differences in infant feeding practices by specific ethnic groups might be lost [24]. Drawing from international findings from the United States and Canada; Vietnamese-born mothers have shorter exclusive breastfeeding durations and increased reliance on infant formulas [25–28]. However, it is unknown whether differences in infant feeding practices exist in Vietnamese-mothers living in Australia.

The aim of this study was to examine the differences in infant feeding practices such as breastfeeding, formula feeding and complementary feeding (i.e., other fluids and solids) in Vietnamese-born mothers compared with Australian-born mothers living in Australia. This knowledge will inform culturally appropriate infant feeding promotion to support optimal health of Vietnamese-Australian children living in Australia.

## Methods

### Study design and participants

This study conducted secondary data analysis of infant feeding data collected by the Australian Institute of Health and Welfare (AIHW) via the Australian National Infant Feeding Survey 2010–11 (ANIFS), a large, national cross-sectional survey measuring the feeding practices of infants aged 0–24 months in Australia [29].

### Australian National Infant Feeding Survey 2010–11 (ANIFS)

Details of the survey methodology have been described elsewhere [29]. In brief, children aged 0–24 months of age were randomly selected Australia wide from the Medicare enrolment database (Australia’s national health care system) [30]. A primary approach letter, reply paid envelope and survey materials were mailed to the primary Medicare cardholders of the infants and completed by mothers/caregivers either online or via reply-paid post [29]. Mothers/caregivers of infants responded to survey questions relating to maternal/child demographics and infant feeding practices (breastfeeding, formula feeding and complementary feeding). Mothers reported if their child had ever been exposed to infant feeding practices (yes/no) and the age of their child when exposure first occurred (months). Survey questions were developed by the Australian Government Department of Health and Ageing and the Australian Bureau of Statistics [29]. Of the 52,008 potential participants invited to participate, 28,759 respondents completed the survey, representing a response rate of 56.4% [29].

### Measures

Maternal self-reported demographic variables included maternal/infant date of birth; postcode; country of birth; main language spoken at home; current smoking status; parity; presence of a spouse/partner pre/post birth; schooling and educational qualifications; total annual gross household income and employment since infant’s birth. Mothers’ ethnicity was determined by self-reported country of birth responses (i.e., Vietnam or Australia). Mother’s age (years) and infant’s age at survey completion (months) were calculated using respective date of birth. Maternal body mass index (BMI) at the start of pregnancy and at survey completion was calculated using self-reported maternal height and weight. Socioeconomic status was defined using postcode as per the Socio-Economic Indexes for Areas Score of Relative Disadvantages (SEIFA) and presented as quintiles [31].

Infant feeding practices examined in this study were related to exposure and timing of exposure to breastfeeding; formula feeding and complementary foods and other fluids (see Supplementary Table 1 for definitions). Mothers reported on exposure to breastmilk; infant formula; water; cow’s milk; toddler milk; soy milk; water-based drinks; fruit juice; and soft, semi-softened and solid foods [29]. Formula milks referred to pre-term formula, infant formula, follow-on formula, soy formula and lactose-free formula but excluded milks suitable for children aged 12 months or more, such as toddler milk drinks which were examined independently [29]. Water included any sips of water and excluded water combined with other fluids or solids (included within water-based drinks) [29]. Cow’s milk and soy milk included any sips of these milks and included flavoured or powdered milks [29]. Water-based drinks included cordial (a non-carbonated sugar sweetened beverage), soft drink, and tea [29]. Soft, semi-solid or solid foods included custards and mashed food diluted with water, milk, or other fluids [29].

### Participants

**Fig. 1:**
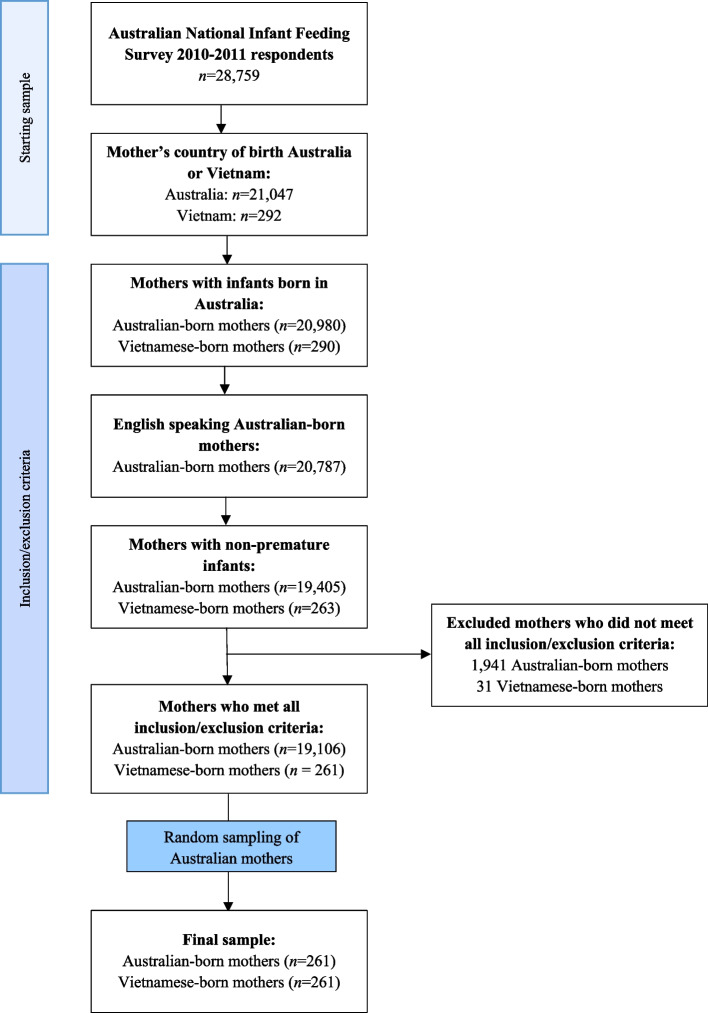
Sampling method of the current study. Note: Ethnicity in this study was defined by a mother’s country of birth. Vietnamese-born mothers refer to mothers who were currently living in Australia but answered ‘Vietnam’ as their country of birth on the Australian National Infant Feeding Survey 2010–2011. Australian-born mothers were mothers currently living in Australia; answered ‘Australia’ as their country of birth and spoke only English at home

Data from two ethnic groups within the ANIFS dataset (Vietnamese-born mothers and Australian-born mothers) that met the following inclusion/exclusion criteria was extracted for analysis. Mothers were included if they (a) answered ‘Australia’ or ‘Vietnam’ as their country of birth in the ANIFS, (b) who had infants born in Australia and (c) who had non-premature infants [32, 33]. Mothers were excluded from the sample if they were non-English speaking Australian-born mothers. The two groups included all Vietnamese-born mothers (*n* = 261) and a randomly selected equal sub-sample of Australian-born mothers (*n* = 261) drawn using the *sample* command in StataSE16 [34] from the 19,106 Australian women that met the inclusion/exclusion criteria (Fig. 1). Two reproducibility analyses were conducted to examine whether the findings were consistent across differently generated samples [35], specifically: 1) all Australian-born mothers in the ANIFS, and 2) a maternal demographically matched Australian-born sample.

### Data preparation and statistical analyses

Australian infant feeding guidelines recommend the introduction of complementary foods at around six months of life [36]. Complementary foods introduced at/prior to four months of life is associated with rapid infant weight gain [37]. Therefore, cut-offs were determined to establish compliance with the Australian infant feeding guidelines (< 6 months and ≥ 6 months) [36] and increased risk of rapid infant weight gain associated with early exposure to complementary foods (≤ 4 months) [37].

Descriptive statistics (means, proportions) were used to summarise each of the demographic and infant feeding practices variables. Independent t-tests and Pearson’s Chi-square tests were used to examine group (i.e., ethnicity group) differences on key demographic variables. Logistic and linear regression analyses were used to test for associations between ethnicity on exposure and timing of infant feeding practices. Separate binary logistic regressions examined the association of ethnicity on exposure to dichotomous infant feeding practice variables (i.e., ever had infant formula (yes/no)). Separate multiple linear regression analyses were performed to examine the association of ethnicity on timing of exposure in continuous infant feeding practice variables (i.e., age of infant when first exposed to infant feeding practice (months)). Regression analyses were adjusted for covariates identified by a literature [38] and included maternal age; SEIFA; parity; maternal BMI at the start of the pregnancy; infant age at survey completion and parity [13, 14, 20–23, 39, 40]. Australian-born mothers were the reference category. Results were summarised using odds ratios, unstandardised (*b*) coefficients, standard error and 95% confidence intervals. All analyses were conducted in StataSE16 [34] with *p* < 0.05 considered a statistically significant finding.

## Results

There were significant group differences for all characteristics, aside from maternal age and parity (Table 1). Compared with Australian-born mothers, a higher proportion of Vietnamese-born mothers experienced more socioeconomic disadvantage; and had a lower annual gross household income. A lower proportion of Vietnamese-born mothers had a partner at the time of their infant’s birth who usually lived in the home; had completed an educational qualification; had achieved a diploma/certificate or higher; were current smokers or had been employed since the birth of their child. Mean BMI was lower at the start of pregnancy and at survey completion for Vietnamese-born mothers. Mean infant age at survey completion was not significantly different between Vietnamese-born and Australian-born mothers.

**Table 1:** Demographic characteristics of Vietnamese-born mothers and Australian-born mothers living in Australia

	**Australian-born mothers** ***n* = 261**	**Vietnamese-born mothers** ***n* = 261**	***P*-value**
	**Mean (*SD*)**	**Mean (*SD*)**	
**Maternal BMI**			
BMI at start of pregnancy	24.2 (4.3)	21.2 (2.5)	< 0.001
BMI at survey completion	25.2 (4.7)	22.5 (2.7)	< 0.001
**Infant age at survey completion**			
Age in months	7.2 (5.6) Range: 1 to 25	7.2 (5.5) Range: 2 to 25	0.981
	**Proportion (%)**	**Proportion (%)**	
**Maternal age**			
15–24 years	7.3	4.6	0.052
25–29 years	25.1	17.0	
30–34 years	34.0	39.0	
35 + years	33.6	39.4	
**Socioeconomic disadvantage (SEIFA) (quintiles) **^a^			
1^st^ quintile (greatest disadvantage)	10.1	50.0	< 0.001
2^nd^ quintile	13.6	12.4	
3^rd^ quintile	22.1	9.7	
4^th^ quintile	24.0	13.6	
5^th^ quintile (least disadvantage)	30.2	14.3	
**Main language**			
English (yes)	100	33.6	< 0.001
**Spouse/partner usually living in the home**			
Yes	95.4	84.6	< 0.001
**Spouse/partner at time of birth**			
Yes	95.4	87.3	0.001
**Completed educational qualification (trade, certificate, bachelor’s degree, diploma, advanced diploma)**			
Educational qualification (yes)	79.2	63.6	< 0.001
**Highest qualification**			
Postgraduate/Bachelor’s degree	40.4	41.5	< 0.001
Diploma/Certificate	36.2	21.5	
Year 12 other	12.7	22.7	
Year 11 other	10.8	14.2	
**Gross household income**			
> $156,000	12.6	9.0	< 0.001
$88,400—$155,999	36.0	18.0	
$52,000—$88,399	27.9	15.7	
$26,000—$51,900	15.0	21.6	
< $25,999	8.5	35.7	
**Parity**			
One	38.7	42.5	0.428
Two	37.2	35.8	
Three	17.8	18.3	
Four or more	6.3	3.3	
**Current smoker**			
Yes	10.3	1.7	< 0.001
**Employment since birth of child**			
Yes	44.0	30.6	0.008

*BMI* Body Mass Index, *SEIFA* Socioeconomic Index for Areas. Gross household income is reported in Australian Dollars (AUD$)

^a^Interpretation of SEIFA: 1^st^ quintile greatest disadvantage, 5^th^ quintile least disadvantage [31]. Ethnicity in this study was defined by a mother’s country of birth. Vietnamese-born mothers refer to mothers who were currently living in Australia but answered ‘Vietnam’ as their country of birth on the Australian National Infant Survey 2010–2011. Australian-born mothers were mothers currently living in Australia; answered ‘Australia’ as their country of birth and spoke only English at home

Table 2 presents descriptive data regarding feeding practices of infants of Vietnamese-born mothers and Australian-born mothers living in Australia. A lower proportion of infants of Vietnamese-born mothers were receiving breastmilk at the time of survey completion, had ever been exposed to soft, semi-solid or solid foods, had been exposed to soft, semi-solid or solid foods prior to six months of age and at/prior to four months of age. Compared with infants of Australian-born mothers, infants to Vietnamese-born mothers were older when they stopped receiving breastmilk and when they were first exposed to soft, semi-solid or solid foods but younger than infants to Australian-born mothers when they were first exposed to fruit juice. A higher proportion of infants of Vietnamese-born mothers had been exposed to infant formula and toddler milks compared with infants of Australian-born mothers.

**Table 2:** Feeding practices of Vietnamese-born mothers and Australian-born mothers living in Australia

		**Infants of Australian-born mothers *n* = 261**		**Infants of Vietnamese-born mothers *n* = 261**	
**Exposure to infant feeding practices**	**Total *n***^a^	***n***	**%**	***n***	**%**
Currently receiving breastmilk (yes)	487	148	60.4	124	51.2
Age stopped breastmilk					
0–6	204	78	82.1	80	73.4
7–12		15	15.8	24	22.0
> 12		2	2.1	5	4.6
Ever drunk infant formula products (yes)	437	170	79.4	189	84.8
Ever drunk water (yes)	426	161	75.9	176	82.2
Ever drunk cow’s milk (yes)	433	40	18.7	35	16.0
Ever drunk toddlers’ milk (yes)	434	4	1.9	35	15.9
Ever drunk soy milk (yes)	434	12	5.6	11	5.0
Ever drunk any water-based drinks (yes)	434	30	14.1	44	19.9
Ever drunk fruit juice (yes)	435	47	22.1	61	27.5
Ever eaten soft, semi-solid, solid foods (yes)	432	142	66.7	119	54.3
Infants introduced to soft, semi-solid, solid foods prior to 6 months (yes)	253	106	75.2	61	54.5
Ever eaten soft, semi-solid, solid foods ≤ 4 months (yes)	253	58	41.1	29	25.9
		**Infants of Australian-born mothers *n* = 261**		**Infants of Vietnamese-born mothers *n* = 261**	
**Timing of exposure to infant feeding practices**	**Total *n***^a^	***n***	**Mean (*SD*)**	***n***	**Mean (*SD*)**
Age stopped receiving breastmilk (months)	204	95	3.23 (4.04)	109	4.87 (4.73)
Age when first drank infant formula products (months)	353	170	1.65 (2.45)	183	1.83 (3.11)
Age when first drank cow’s milk (months)	73	38	10.61 (2.52)	35	12.97 (7.14)
Age when first drank soy milk (months)	20	11	9.45 (8.04)	9	11.56 (5.05)
Age when first drank water-based drinks (months) ^b^	70	26	9.54 (7.15)	44	7.75 (6.88)
Age when first drank fruit juice (months)	107	48	10.58 (5.68)	59	7.39 (4.87)
Age when first ate soft, semi-solid, solid foods (months)	253	141	4.76 (1.05)	112	5.53 (1.88)

Timing of exposure to water and toddler milks were not measured in the Australian National Infant Feeding Survey 2010–2011, only whether the infant was exposed to these fluids

^a^Varying total sample sizes were a result of mothers not answering all survey questions or the age of their infant dictated whether they had been exposed to that infant feeding practice

^b^Water-based drinks included cordial, soft drink and tea

Table 3 presents the results of the logistic and linear regressions analyses. In unadjusted analyses, compared with infants of Australian-born mothers, ethnicity was positively associated with exposure to toddler milks; and negatively associated with whether the infant was receiving breastmilk at the time of survey completion and had been exposed to soft, semi-solid or solid foods (ever, prior to 6 months and prior to 4 months) (*p* < 0.05). Infants of Vietnamese-born mothers were older when they stopped receiving breastmilk and when first exposed to soft, semi-solid or solid foods; but younger when they were first exposed to fruit juice than infants to Australian-born mothers (*p* < 0.05).

**Table 3:** Regression analyses examining the association of ethnicity on infant feeding practices in Vietnamese-born and Australian-born mothers

**Binary logistic regression**^a^	**Unadjusted **^b^				**Adjusted**^c^			
Variable	***n***^d^	**OR**	**95% CI**	***P*-value**	***n***	**OR**	**95% CI**	***P*-value**
Infant currently receiving breastmilk? (yes)	487	0.68	0.48 – 0.99	0.042	414	0.60	0.35 – 1.02	0.061
Infant ever had formula? (yes)	437	1.44	0.88 – 2.36	0.148	367	2.21	1.10 – 4.43	0.025
Infant ever had cow’s milk? (yes)	433	0.83	0.50—1.36	0.457	364	1.11	0.38 – 3.15	0.848
Infant ever had water? (yes)	426	1.46	0.92 – 2.35	0.111	360	1.24	0.64 – 2.41	0.525
Infant ever had toddler milk? (yes)	434	9.93	3.46 – 28.4	< 0.001	365	16.72	3.11 – 90.09	0.001
Infant ever had soy milk? (yes)	434	0.88	0.37—2.03	0.760	303	1.14	0.29 – 4.45	0.851
Infant ever had any water-based drinks? (yes)	434	1.52	0.91 – 2.52	0.108	364	1.46	0.66 – 3.23	0.347
Infant ever had fruit juice? (yes)	435	1.34	0.86 – 2.07	0.192	365	2.37	1.06 – 5.32	0.037
Infant ever had solids? (yes)	432	0.60	0.40 – 0.88	0.009	362	0.15	0.05 – 0.44	0.001
Given solids ≤ 4 months? (yes)	253	0.5	0.29 – 0.86	0.012	210	0.70	0.32 – 1.49	0.353
Given solids < 6 months? (yes)	253	0.39	0.23 – 0.67	0.001	210	0.63	0.27 – 1.45	0.274
**Multiple linear regression**^a^	**Unadjusted** ^b^				**Adjusted**^c^			
Variable	***n***^d^	***b* (*SE*)**	**95% CI**	***P*-value**	***n***	***b* (*SE*)**	**95% CI**	***P*-value**
Age stopped receiving breastmilk (months)	204	1.64 (0.62)	0.42 – 2.86	0.009	172	1.39 (0.75)	-0.10 – 2.87	0.067
Age when first drank infant formula products (months)	353	0.17 (0.30)	-0.42 – 0.76	0.566	298	0.08 (0.35)	-0.60 – 0.76	0.821
Age when first drank cow’s milk products (months)	73	2.37 (1.23)	-0.09 – 4.82	0.059	62	1.36 (0.89)	-0.43 – 3.15	0.134
Age when first drank soy milk (months)	20	2.10 (3.09)	-4.39 – 8.59	0.505	16	2.68 (0.47)	-6.07 – 11.42	0.467
Age when first drank water-based drinks (months)	70	-1.79 (1.73)	-5.24 – 1.66	0.304	57	-1.70 (1.56)	-4.85 – 1.44	0.281
Age when first drank fruit juice (months)	107	-3.19 (1.02)	-5.22 – -1.17	0.002	84	-2.41 (1.07)	-4.54 – -0.28	0.027
Age when first ate soft, semi-solid, solid foods (months)	253	0.77 (0.19)	0.40 – 1.14	< 0.001	210	0.41 (0.24)	-0.06 – 0.87	0.085

Separate logistic and linear regressions were undertaken for each variable presented in this table

*b* unstandardised beta coefficient, *CI* confidence intervals, *n* sample size, *SE* standard error, *OR* odds ratio

^a^Reference category for regressions: *Australian-born mothers*

^b^Unadjusted: *Ethnicity on infant feeding practices*

^c^Adjusted: *Maternal age, SEIFA, BMI at start of pregnancy, infant age at survey completion and parity*

^d^Varying sample sizes across regressions were a result of mothers not answering all survey questions or the age of their infant dictated whether they had been exposed to that infant feeding practices

In adjusted logistic regression analyses, ethnicity was significantly positively associated with ever exposed to formula, toddler milks and fruit juice. Infants of Vietnamese-born mothers had 2.21 times higher odds of ever receiving formula; 2.37 times higher odds of ever receiving fruit juice and almost 17 times higher odds of ever receiving toddler milk relative to infants of Australian-born mothers (*p* < 0.05). Ethnicity was negatively associated with exposure to soft, semi-solids and solid foods; infants of Vietnamese-born mothers had 85% lower odds of ever being exposed to soft, semi-solids and solids relative to infants of Australian-born mothers (*p* = 0.001). Adjusted linear regression analyses revealed ethnicity to be associated with timing of exposure to fruit juice, meaning a one unit increase in ethnicity (from Australian-born to Vietnamese-born) was associated with a 2.41 month decrease in age when infants were exposed to fruit juice (*p* = 0.027). The reproducibility analysis on all Australian-born mothers; and the demographically matched Australian-born sample produced similar findings (Supplementary Tables 2 and 3).

## Discussion

This study comprehensively examined the infant feeding practices of Vietnamese-born mothers living in Australia in isolation to other Asian ethnic groups. This is important given the lack of available data on the infant feeding practices in this immigrant group which is the fifth largest migrant group in Australia. Examining this immigrant group in isolation allows for a better understanding of specific nuances which might be lost when combined with broader South-East Asian and Asian groups.

Low adherence to breastfeeding and other fluids recommendations outlined by the Australian infant feeding guidelines [36] were observed in this group of Vietnamese-born mothers. Specifically, infants of Vietnamese-born mothers were less likely to have been exposed to solids and more likely to have been exposed to infant formula, toddler milks and fruit juice when compared with infants of Australian-born mothers. These findings may have important implications for the healthy growth and development of Australian-Vietnamese children living in Australia [2].

There was low compliance with Australian infant feeding guidelines by both Vietnamese-born and Australian-born mothers. Not breastfeeding an infant until 12 months of age may reduce the protective effect of breastfeeding  on reducing risk of overweight and obesity in infants [41–43]. The low compliance with breastfeeding until 12 months may be explained by infant formula feeding or a mixed feeding approach (i.e., breastmilk in combination with infant formula). Whilst Vietnamese-born mothers in this study demonstrated higher breastfeeding exposure at survey completion (when infants were on average, seven months old) than previously reported in Australia [44]; overall breastfeeding practices were suboptimal which is consistent with studies in the United States, Canada and the United Kingdom [25–28]. These studies should be interpreted with caution given various breastfeeding definitions and terminology (e.g. currently breastfed, exclusive breastfeeding, breastfeeders, non-breastfeeders) and the data is outdated. Similar to a study examining Chinese ethnicity on breastfeeding practices [19], Vietnamese ethnicity was not associated with exposure to breastmilk or the timing of breastmilk cessation. Despite both studies using a national sample, a plausible explanation for these consistent findings may have been the inability to adjust for timing of exposure to formula milks and complementary foods, which is considered a predictor of breastfeeding duration [13]; along with complex sociodemographic, physical, mental and social factors which may influence infant feeding practices such as maternal working status, breastfeeding knowledge, delivery mode, parity, maternal infant feeding attitude, intention, baby behaviours (fussiness, crying), lactation problems (milk insufficiency, maternal breastfeeding confidence), and introduction of formula [45–47]. Future studies should consider these in the study design, data collection and analysis.

The current study identified infants of Vietnamese-born mothers were twice as likely to have been exposed to infant formula compared with their Australian-born counterparts. Given formula feeding is associated with an increased risk of rapid weight gain during infancy driven by the high protein and energy content (in metabolic excess) of infant formula compared to breastmilk [7, 11, 48]. Other factors such as the size of the bottle, going to bed with a bottle of formula, parental feeding practices (feeding on demand or schedule; bottle emptying), formula preparation (e.g. overconcentration) may also influence rapid weight gain [11]. This is an ideal behaviour to target if implementing health promotion strategies. Vietnamese-born mothers have been reported to adopt formula feeding after immigration to higher socioeconomic countries including the United States [26], Canada [28], and Australia [49, 50]. This is also true for Chinese-born mothers [19]. There are several possible explanations for these similarities. Infant feeding practices in Asian ethnic mothers are embedded in cultural beliefs and perceptions [24, 51]. Like Chinese-born mothers living in Australia, Vietnamese-born mothers report beliefs that formula milks are a healthier western option [24, 52]. Emerging evidence suggests that formula feeding is also becoming a norm in Vietnam itself [53]. Additionally, specific Vietnamese postnatal rituals affirm a mothers’ ability to breastfeed effectively [24]. If mothers felt that they were unable to partake in postnatal rituals after immigration, their maternal capacity to breastfed was negatively impacted, resulting in an increased reliance on infant formula [24, 51]. Additionally, a lack of awareness of the Australian infant feeding guidelines (or appropriate translations that are readily accessible and culturally sensitive) or poorer access/awareness of post-natal breastfeeding services may increase reliance on formula [36, 54–56]. In Vietnamese mothers living in Vietnam, those who had better awareness of infant feeding recommendations and breastfeeding support by a health worker demonstrated improved breastfeeding exclusivity duration and reduced reliance on infant formula [57]. In one Australian study, the majority of mothers surveyed experienced difficulties communicating with health professionals due to language barriers, a lack of positive attitudes towards breastfeeding health professionals and a lack of social and family support postally to influence breastfeeding duration [50]. Future qualitative studies understanding the barriers and enablers to breastfeeding and formula feeding in this ethnic group would be beneficial.

A higher proportion of Vietnamese-born mothers adhered to complementary feeding recommendations for solids [36] (54.5% of infants of Vietnamese-born mothers vs 75.2% of Australian-born infants were introduced to solids before 6 months of age). This finding is consistent with Chinese-born and Indian-born mothers [19, 58]. Evidence from studies in Asian ethnic mothers suggests that a range of factors may influence timing of exposure to solids such as by cultural or ethnic perceptions of infant readiness for food [24], the presence of infant teeth [24] and the prevention of allergies [59]. Infants of Vietnamese-born mothers in this study were older (5.53 months) when first exposed to solids than infants of Australian-born mothers (4.76 months) and older reports in Vietnamese infants (4.4 months) [44]. No association between ethnicity on timing of exposure to solids was observed in this study which is inconsistent that Vietnamese-born mothers were less likely to expose their infants to solids very early (≤ 4 months of age) compared with Australian-born mothers [14]. Inconsistencies across studies may be due to adjustment for feeding method at four weeks, which has been identified as a significant predictor of early introduction to solids [14]. Additionally, the study also defined very early introduction as before four months (< 4 months) whilst the current study was inclusive of four months of age (≤ 4 months).

Ethnicity was positively associated with exposure to toddler milk in infants of Vietnamese-born mothers within this study. Infants of Vietnamese-born mothers had higher odds of exposure to toddler milk compared with infants of Australian-born mothers; a finding that is inconsistent with Australian infant feeding guidelines which state that toddler milks are not recommended for optimal growth. Australian toddler milks are identified to contain added sugars, nearly as high as some soft drinks [60] and may also provide excess energy and protein requirements [7]. This may place Vietnamese-Australian children at a greater risk of rapid weight gain trajectories and developing sweet taste preferences [61]. Examination of toddler milk consumption in Vietnamese-born mothers is limited both nationally in Australia, and internationally; and it is unknown what drives this feeding practice. Drawing from perceptions of infant formula use, perhaps the same belief that infant formula is a western and modern alternative is extended to include toddler milks [24], and like formula possibly becoming a norm [53]. It is also possible that the historical concerns of undernutrition may influence the use of infant formulas and toddler milks. Marketing tactics regarding ultra processed milk formulas which contain high levels of sugar for pregnant women in Vietnam may persuade mothers to continue with other products during infancy and early childhood due to beliefs that these products can make a child healthy and smart [53]. Analysis of sales data on infant/child milk-based formulas has revealed growth to be most rapid in East Asia, including Vietnam [62]. Inappropriate marketing from industry is a great concern and more needs to be done to ensure marketing tactics don’t violate the regulatory codes such as the International Code of Marketing Breast-Milk Substitutes which was established to protect and promote optimal infant and young child feeding [62, 63]. These marketing influences may migrate with mothers coming to Australia. Further research is needed to confirm these hypotheses.

This study is also the first report high exposure to fruit juice, and exposure at an earlier age in infants of Vietnamese-born mothers in Australia. This is similar to infants of Chinese-born and Indian-born mothers living in Australia [19, 58]. The Australian infant feeding guidelines do not recommend fruit juice before 12 months of age [36] due to the negative consequences of excess added sugars on infant health such as increased risk of dental caries [64]. Delaying the introduction of sweets and fruit juice may be associated with less consumption of these foods and higher diet quality later in life [61, 65, 66]. A similar study conducted in Sydney revealed Vietnamese-born mothers were twice as likely to introduce sugar sweetened beverages (including fruit juice and water-based drinks) prior to 12 months of age [23]. Taken together, culturally appropriate education on toddler milks and fruit juice for Vietnamese-born mothers needs to be prioritised to optimise infant growth and health.

Strengths of this study include the analysis of the largest sample of Vietnamese-born immigrants living in Australia to date and the use of a comprehensive national dataset allowing for generalisability. A wide range of infant feeding practices were examined in Vietnamese-born mothers in isolation, rather than combined with a larger Asian ethnic group which may have differing cultures and beliefs and are underrepresented in the literature; and data was able to be adjusted for a range of maternal and infant covariates. Furthermore, two reproducibility analyses were conducted to examine the risk of the reported results being idiosyncratic to the analysed sample. The reproducibility analysis on the two samples derived from different methods were remarkedly similar, and taken together they ensure trustworthiness of the study and strengthen the conclusions [35]. Several study limitations are acknowledged. Interpretation of the findings are limited by the cross-sectional nature of the study. The ANIFS was a self-report survey which is subject to social-desirability bias and recall bias. Additionally, although non-English speaking Vietnamese-born mothers responded to the ANIFS, the survey was sent out in English potentially excluding some mothers who were not linguistically diverse. This study was unable to adjust for confounding factors including father’s ethnicity, level of acculturation, length of residency and level of social support in Australia, which may have impacted infant feeding practices. Although the most recent, the data is limited to findings from 2010–2011. More contemporary data is required to reflect current mothers living in Australia.

## Conclusions

Differences in infant feeding practices exist amongst Vietnamese-born and Australian-born mothers living in Australia. This study has identified key infant feeding practices of focus for future health promoting strategies to support Vietnamese-born mothers to feed their infants for optimal growth and health. Focusing on supporting breastfeeding for a longer duration, less reliance on infant formula and reducing exposure to unnecessary other fluids such as toddler milk and fruit juice is required. Given the expected growth of the Australian immigrant population, culturally sensitive and accessible support ensuring optimal nutrition within the first one thousand days of life is imperative to the long-term health of infants of Vietnamese-born immigrant mothers. In conjunction with a contemporary understanding of the drivers of infant feeding practices, including cultural perceptions, beliefs, and traditions, awareness of the infant feeding guidelines, level of social support and engagement or accessibility to infant feeding support; there is a need to implement and evaluate culturally appropriate initiatives to encourage optimal infant feeding practices.

## Supplementary Information

**Additional file 1: Supplementary Table 1.** Infant feeding practices measured in the current study.

**Additional file 2: Supplementary Table 2.** Sample demographics for reproducibility analysis. **Supplementary Table 3.** Reproducibility regression analyses examining the association of ethnicity on infant feeding practices in Vietnamese-born and Australian-born mothers.

## Data Availability

In 2010–2011 The Australian Institute of Health and Welfare (AIHW) conducted The Australian National Infant Feeding Survey (ANIFS) (29). The data used within this study is publicly available upon request by the data custodian (Australian Data Archive, https://ada.edu.au/).

## Abbreviations

ABS: Australian Bureau of Statistics
ADG: Australian Dietary Guidelines
AIHW: Australian Institute of Health and Welfare
ANIFS: Australian National Infant Feeding Survey 2010–2011
AOR: Adjusted Odds Ratio
*b*: Unadjusted Beta Coefficient
BMI: Body Mass Index
CI: Confidence Intervals
NHMRC: National Health and Medical Research Council
OR: Odds Ratio
*p*: *P*-Value
SE: Standard Error
SEIFA: Socio-Economic Indexes For Areas Score of Relative Disadvantages
SD: Standard Deviation
UNICEF: United Nations Children’s Fund
WHO: World Health Organization
