# Supplementary material for: A cross-sectional study of infant feeding practices in Vietnamese-born mothers living in Australia
Source: BMC Pregnancy Childbirth. 2022 Dec 3;22:895. doi: 10.1186/s12884-022-05223-8 (PMC9719657; doi:10.1186/s12884-022-05223-8)
Supplement: Supplementary file 1 — Additional file 1: Supplementary Table 1. Infant feeding practices measured in the current study. [file 12884_2022_5223_MOESM1_ESM.docx]

Supplementary Table 1: Infant feeding practices measured in the current study

| Variable | ANIFS question | Measurement | Definition^a^ |
| --- | --- | --- | --- |
| Breastfeeding |  |  |  |
| Exposure to breastmilk | ‘Is your child currently receiving breast milk?’. | Yes/No | Breastmilk included colostrum, expressed breast milk and breast milk from a donor or donor milk bank. |
| Breastfeeding duration | ‘How old was your child when he/she stopped receiving any breast milk?’. | Childs age in months |  |
| Formula feeding |  |  |  |
| Exposure to infant formula | ‘Has your child ever drunk any infant formula products?’. | Yes/No | Formula milks referred to pre-term formula, infant formula, follow-on formula, soy formula and lactose-free formula. But excluded milks suitable for children aged 12 months or more, such as toddler milk drinks. |
| Timing of exposure to infant formula | ‘How old was your child when he/she first drank an infant formula product?’. | Childs age in months |  |
| Complementary feeding: solids | | | |
| Exposure to complementary foods | ‘Has your child ever eaten any soft, semi-solid or solid foods?’. | Yes/No | Complementary foods referred to soft, semi-solid or solid foods and included custards, mashed food diluted with water, milk, or other fluids. |
| Timing of exposure to complementary foods | ‘How old was your child when he/she first ate soft, semi-solid or solid foods?’. | Childs age in months |  |
| Complementary feeding: liquids | | | |
| Exposure to complementary liquids | ‘Has your child ever drunk water/cow’s milk/toddler milk/soy milk/water-based drinks/fruit juice/other fluids?’. | Yes/No | Complementary liquids included water, cow’s milk, soy milk, water-based drinks and fruit juice.  Water included any sips of water and excluded water combined with other liquids or solids.  Cow’s milk and soy milk included any sips of these milks and included flavoured or powdered milks.  Water based drinks included cordial, soft drink and tea.  Diluted fruit juice and infant formula products were excluded. |
| Timing of exposure to complementary liquids | ‘How old was your child when he/she first drank cow’s milk/soy milk/water-based drinks/fruit juice?’. | Childs age in months |  |

**Note:** Although *exposure* to water and toddler milks were measured by the ANIFS, *timing of exposure* (age in months) to water and toddler milks were not (30).

^a^Definitions outlined by the Australian National Infant Feeding Guidelines (4, 36). ANIFS: Australian National Infant Feeding Survey 2010-2011.
