# Supplementary material for: A cross-sectional study of infant feeding practices in Vietnamese-born mothers living in Australia
Source: BMC Pregnancy Childbirth. 2022 Dec 3;22:895. doi: 10.1186/s12884-022-05223-8 (PMC9719657; doi:10.1186/s12884-022-05223-8)
Supplement: Supplementary file 2 — Additional file 2: Supplementary Table 2. Sample demographics for reproducibility analysis. Supplementary Table 3. Reproducibility regression analyses examining the association of ethnicity on infant feeding practices in Vietnamese-born and Australian-born mothers. [file 12884_2022_5223_MOESM2_ESM.docx]

***Supplementary Table 2 Sample demographics for reproducibility analysis***

|  | Vietnamese-born mothers only  *n*= 261 | Random sample of  Australian-born mothers  *n*= 261 | All sample  Australian-born mothers  *n*= 19,106 | Age, parity, SEIFA matched  Australian-born mothers  n= 261 |
| --- | --- | --- | --- | --- |
|  | **Proportion (%)** | **Proportion (%)** | **Proportion (%)** | **Proportion (%)** |
| Maternal age | | | | |
| 15-24 years | 4.6 | 7.3 | 10.3 | 4.6 |
| 25-29 years | 17.0 | 25.1 | 25.3 | 17.0 |
| 30-34 years | 39.0 | 34.0 | 35.5 | 39.0 |
| 35+ years | 39.4 | 33.6 | 28.9* | 39.4 |
| Socioeconomic disadvantage (SEIFA) (quintiles) ^a^ | | | | |
| 1^st^ quintile (greatest disadvantage) | 50.0 | 10.1 | 11.8 | 50.0 |
| 2^nd^ quintile | 12.4 | 13.6 | 17.3 | 12.4 |
| 3^rd^ quintile | 9.7 | 22.1 | 21.8 | 9.7 |
| 4^th^ quintile | 13.6 | 24.0 | 23.7 | 13.6 |
| 5^th^ quintile (least disadvantage) | 14.3 | 30.2 | 25.5* | 14.3 |
| Parity | | | | |
| One | 42.5 | 38.7 | 41.8 | 42.5 |
| Two | 35.8 | 37.2 | 36.7 | 35.8 |
| Three | 18.3 | 17.8 | 15.2 | 18.3 |
| Four or more | 3.3 | 6.3 | 6.3* | 3.3 |

****p<0.05***

***Supplementary Table 3: Reproducibility regression analyses examining the association of ethnicity on infant feeding practices in Vietnamese-born and Australian-born mothers.***

***Analysis 1 all Vietnamese-born vs random Australian-born subsample***

***Analysis 2 all Vietnamese-born vs all Australian-born subsample***

***Analysis 3 all Vietnamese-born vs age, parity, SEIFA matched Australian-born subsample***

| Binary logistic regression | *Vietnamese vs random sample* | | | | *Vietnamese vs all Australian sample* | | | | *Vietnamese vs age, parity, SEIFA matched* | | | |
| --- | --- | --- | --- | --- | --- | --- | --- | --- | --- | --- | --- | --- |
| Variable | ***n*** | **OR** | **95% CI** | ***p*-value** | ***n*** | **OR** | **95% CI** | ***p*-value** | ***n*** | **OR** | **95% CI** | ***p*-value** |
| Infant currently receiving breastmilk? (yes) | 414 | 0.60 | 0.35 – 1.02 | 0.061 | 15954 | 0.60 | 0.44-0.81 | 0.001 | 404 | 0.48 | 0.29-0.80 | 0.005 |
| Infant ever had formula? (yes) | 367 | 2.21 | 1.10 – 4.43 | 0.025 | 12863 | 1.89 | 1.25-2.88 | 0.003 | 348 | 2.01 | 1.09-3.70 | 0.024 |
| Infant ever had cow’s milk? (yes) | 364 | 1.11 | 0.38 – 3.15 | 0.848 | 13535 | 0.63 | 0.28-1.39 | 0.251 | 370 | 0.86 | 0.35-2.10 | 0.733 |
| Infant ever had water? (yes) | 360 | 1.24 | 0.64 – 2.41 | 0.525 | 13528 | 2.04 | 1.36-3.08 | 0.001 | 404 | 1.66 | 0.91-3.04 | 0.101 |
| Infant ever had toddler milk? (yes) | 365 | 16.72 | 3.11 – 90.09 | 0.001 | 13526 | 7.2 | 4.25-12.15 | <0.001 | 369 | 9.51 | 3.10-29.23 | <0.001 |
| Infant ever had soy milk? (yes) | 303 | 1.14 | 0.29 – 4.45 | 0.851 | 13542 | 1.70 | 0.84-3.45 | 0.614 | 371 | 1.56 | 0.48-5.08 | 0.456 |
| Infant ever had any water-based drinks? (yes) | 364 | 1.46 | 0.66 – 3.23 | 0.347 | 13353 | 1.59 | 1.00-2.51 | 0.047 | 371 | 1.99 | 0.96-4.11 | 0.065 |
| Infant ever had fruit juice? (yes) | 365 | 2.37 | 1.06 – 5.32 | 0.037 | 13533 | 2.23 | 1.44-3.45 | <0.001 | 372 | 2,46 | 1.18-5,14 | 0.016 |
| Infant ever had solids? (yes) | 362 | 0.15 | 0.05 – 0.44 | 0.001 | 13513 | 0.22 | 0.13-0.37 | <0.001 | 369 | 0.25 | 0.11-0.56 | 0.001 |
| Given solids ≤ 4 months? (yes) | 210 | 0.70 | 0.32 – 1.49 | 0.353 | 8864 | 0.61 | 0.38-0.98 | 0.041 | 218 | 0.63 | 0.33-1.21 | 0.167 |
| Given solids < 6 months? (yes) | 210 | 0.63 | 0.27 – 1.45 | 0.274 | 8854 | 0.52 | 0.33-0.84 | 0.007 | 218 | 0.58 | 0.29-1.14 | 0.114 |
| **Multiple linear regression** | ***Vietnamese vs random sample*** | | | | ***Vietnamese vs all Australian sample*** | | | | ***Vietnamese vs age, parity, SEIFA matched*** | | | |
| Variable | ***n*** | ***b* (*SE*)** | **95% CI** | ***p*-value** | ***n*** | ***b* (*SE*)** | **95% CI** | ***p*-value** | ***n*** | ***b* (*SE*)** | **95% CI** | ***p*-value** |
| Age stopped receiving breastmilk (months) | 172 | 1.39 (0.75) | -0.10 – 2.87 | 0.067 | 6247 | 1.01 (0.33) | 0.41-1.7 | 0.001 | 170 | 1.88 (0.69) | 0.51-3.25 | 0.007 |
| Age when first drank infant formula products (months) | 298 | 0.08 (0.35) | -0.60 – 0.76 | 0.821 | 10775 | -0.02 (0.18) | -0.38-0.34 | 0.922 | 300 | 0.57 (0.34) | -0.09-1.24 | 0.091 |
| Age when first drank cow’s milk products (months) | 62 | 1.36 (0.89) | -0.43 – 3.15 | 0.134 | 2486 | 1.03 (0.45) | 0.15-1.92 | 0.022 | 72 | 1.27 (0.64) | -0.01-2.55 | 0.052 |
| Age when first drank soy milk (months) | 16 | 2.68 (0.47) | -6.07 – 11.42 | 0.467 | 415 | 0.77 (1.40) | -1.97-3.52 | 0.582 | 16 | 2.10 (2.39) | -3.10-7.31 | 0.395 |
| Age when first drank water-based drinks (months) | 57 | -1.70 (1.56) | -4.85 – 1.44 | 0.281 | 1989 | -0.60 (0.61) | -1.80-0.600 | 0.326 | 69 | -1.12 (1.22) | -3.56-1.33 | 0.364 |
| Age when first drank fruit juice (months) | 84 | -2.41 (1.07) | -4.54 – -0.28 | 0.027 | 2632 | -1.77 (0.49) | -2.73—0.80 | <0.001 | 90 | -1.90 (0.93) | -3.75—0.04 | 0.045 |
| Age when first ate soft, semi-solid, solid foods (months) | 210 | 0.41 (0.24) | -0.06 – 0.87 | 0.085 | 8864 | 0.63 (0.12) | 0.39-0.86 | <0.001 | 218 | 0.47 (0.22) | 0.03-0.91 | 0.079 |
